# Supplementary material for: On the analysis of two-time correlation functions: equilibrium versus non-equilibrium systems
Source: J Appl Crystallogr. 2024 Jul 4;57(Pt 4):1098–106. doi: 10.1107/S1600576724004618 (PMC11299609; doi:10.1107/S1600576724004618)
Supplement: Supplementary file 1 [file j-57-01098-sup1.pdf]

JOURNAL OF APPLIED CRYSTALLOGRAPHY

Volume 57 (2024)

Supporting information for article:

On the analysis of two-time correlation functions: equilibrium versus non-equilibrium systems

Anastasia Ragulskaya, Vladimir Starostin, Fajun Zhang, Christian Gutt and Frank Schreiber

S1. *Parameters of simulation*

The simulation is performed on a 2D grid 512×512 for 100 time steps with $\Delta t = 1$ for a set of 512 non-touching particles. The boundary conditions and the particle-particle interactions follow the implementation in (Ragulskaya *et al.*, 2022). Specifi-cally, we avoid interactions with boundaries and other particles by setting appropriate size parameters and initial particle coordinates. In rare cases when such interactions occur, the simulation is terminated and repeated with other initial positions of particles. For each particle the probability density function was defined via Equation (17). The speckle pattern (i.e. image in reciprocal space $I(\mathbf{q},\ t_{age})$) can be calculated for each time step as a square of the magnitude of the 2D fast Fourier transform of the fluctuations of the concentration (Barton *et al.*, 1998). The outcome is similar to the 2D scattering pattern obtained via XPCS experiments. This pattern was then used to calculate the TTC for the simulation via Equation (2).

S2. *Functions and their relations*

- **"*Corr*-TTC"** - calculation of the TTC via *Corr* (see Equation (1)) - the normalization to the mean intensity.
- **"*G*-TTC"** - calculation of the TTC via G (see Equation (2)) - the normalization to the standard deviation of the intensity.

	ACS	CCS
g_1	$g_{1_{ACS}}(t,\Delta t) = \langle E^*(t-\Delta t/2)E(t+\Delta t/2)\rangle_N$ Equation (8)	$g_{1_{CCS}}(t,\Delta t) = \langle E^*(t)E(t+\Delta t)\rangle_N$ Equation (7)

Table S1. *Definition of g_1-function for ACS and CCS cuts.*

	G (Equation (2))	$Corr$ (Equation (1))
g_2	$g_{2_G} = \lvert g_1(t,\Delta t)\rvert^2$ Equation (6)	$g_{2_{Corr}} = 1+\beta(\vec{q})\lvert g_1(t,\Delta t)\rvert^2$ Equation (5)
$g_{2_{ACS}}$	$g_{2_{G,ACS}} = \lvert g_{1_{ACS}}(t,\Delta t)\rvert^2$	$g_{2_{Corr,ACS}} = 1+\beta(\vec{q})\lvert g_{1_{ACS}}(t,\Delta t)\rvert^2$
$g_{2_{CCS}}$	$g_{2_{G,CCS}} = \lvert g_{1_{CCS}}(t,\Delta t)\rvert^2$	$g_{2_{Corr,CCS}} = 1+\beta(\vec{q})\lvert g_{1_{CCS}}(t,\Delta t)\rvert^2$

Table S2. *Definition of g_2-function, extracted from G and C-TTCs (first and second column, respectively): in general and for ACS and CCS cuts (first, second and third rows, respectively) in case of valid Siegert relation.*

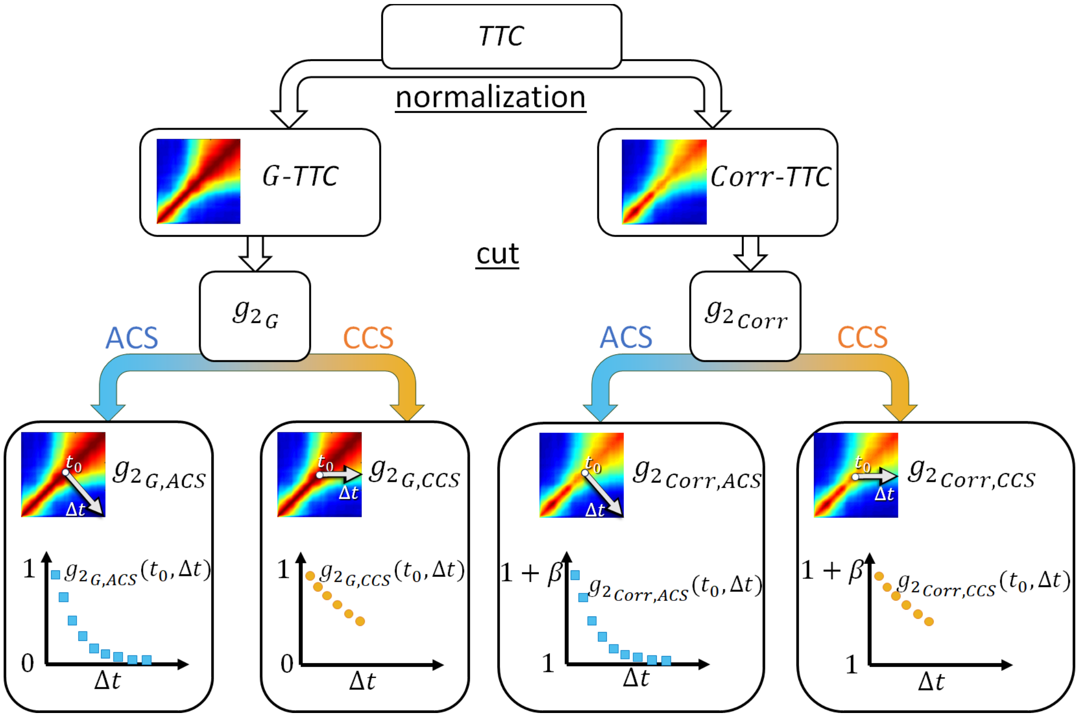

Fig. S1. Scheme of relations between different functions, used in XPCS data analysis.

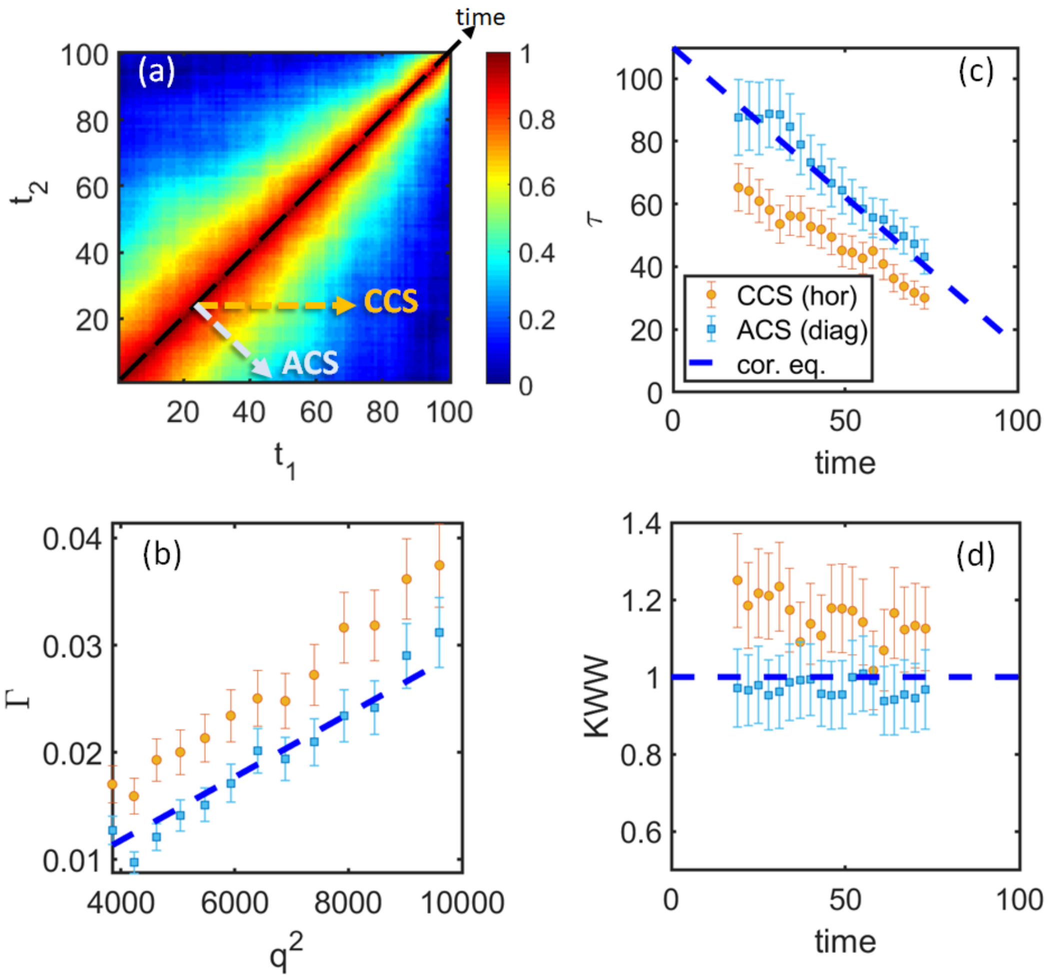

Fig. S2. Data analysis for a model system for *Case 1b* - a linear decrease in $1/D$ (to be compared with Figure 3). (a) G-TTC for $q = 77$ pixel. (b) Relaxation rate Γ as a function of q^2 at $time = 55$. (c) and (d) represent relaxation time τ and KWW as functions of time, correspondingly. Orange circles display results for CCS analysis, light blue squares - for ACS, and dashed blue line shows results from corresponding equilibrium systems. Results for other q and $time$ values are similar.
